# Reading Mammal Diversity from Flies: The Persistence Period of Amplifiable Mammal mtDNA in Blowfly Guts (*Chrysomya megacephala*) and a New DNA Mini-Barcode Target

**DOI:** 10.1371/journal.pone.0123871

**Published:** 2015-04-21

**Authors:** Ping-Shin Lee, Kong-Wah Sing, John-James Wilson

**Affiliations:** 1 Museum of Zoology, Institute of Biological Sciences, Faculty of Science, University of Malaya, 50603 Kuala Lumpur, Malaysia; 2 Ecology and Biodiversity Program, Institute of Biological Sciences, Faculty of Science, University of Malaya, 50603 Kuala Lumpur, Malaysia; Onderstepoort Veterinary Institute, SOUTH AFRICA

## Abstract

Most tropical mammal species are threatened or data-deficient. Data collection is impeded by the traditional monitoring approaches which can be laborious, expensive and struggle to detect cryptic diversity. Monitoring approaches using mammal DNA derived from invertebrates are emerging as cost- and time-effective alternatives. As a step towards development of blowfly-derived DNA as an effective method for mammal monitoring in the biodiversity hotspot of Peninsular Malaysia, our objectives were (i) to determine the persistence period of amplifiable mammal mtDNA in blowfly guts through a laboratory feeding experiment (ii) to design and test primers that can selectively amplify mammal *COI* DNA mini-barcodes in the presence of high concentrations of blowfly DNA. The persistence period of amplifiable mammal mtDNA in blowfly guts was 24 h to 96 h post-feeding indicating the need for collecting flies within 24 h of capture to detect mammal mtDNA of sufficient quantity and quality. We designed a new primer combination for a *COI* DNA mini-barcode that did not amplify blowfly DNA and showed 89% amplification success for a dataset of mammals from Peninsular Malaysia. The short (205 bp) DNA mini-barcode could distinguish most mammal species (including separating dark taxa) and is of suitable length for high-throughput sequencing. Our new DNA mini-barcode target and a standardized trapping protocol with retrieval of blowflies every 24 h could point the way forward in the development of blowfly-derived DNA as an effective method for mammal monitoring.

## Introduction

According to the Global Mammal Assessment (2008), information on the distribution and abundance of most tropical mammal species remains data-deficient, and thus some species appear to be disproportionately threatened [1]. Lack of data on tropical mammal species can be associated with limitations of the existing monitoring approaches. Field-trapping techniques vary in efficiency [2] and are often a biased representation of diversity [3,4]. For example, identification of animal signs is laborious, requiring the input of specialists over an extended time period [5], and can be imprecise [6,7]. Likewise, expensive camera traps cannot identify individuals to species lacking easily observed diagnostic markings [8,9]. Impediments also include the challenge posed by cryptic species, which are not always morphologically distinct, and are more easily recognised by molecular techniques [10,11]. Considering that the current monitoring approaches are challenged by ethics [12], precision, and accuracy, a new approach is urgently needed.

Approaches deriving vertebrate DNA from associated invertebrate taxa are emerging as powerful tools for large-scale monitoring [13]. Such approaches have the potential to detect and identify rare and cryptic mammal species [14,15]. Two rare species—the Annamite striped rabbit (*Nesolagus timminsi*) and the Truong Son muntjac (*Muntiacus truongsonensis*)—as well as a “cryptic” species, the small-toothed ferret-badger (*Melogale moschata*), were detected from bloodmeals recovered from leeches collected in a forest in Vietnam [15].

Blowfly-derived DNA may present advantages over DNA recovered from other invertebrates, such as leeches, which are habitat restricted [15], or mosquitoes and tsetse flies, which have narrow host preferences [16,17]. Blowflies (Calliphoridae) are globally distributed in all habitats and exhibit broad host preferences both during larval and adult stages [18,19]. For example, *Chrysomya megacephala* is a saprophagous and coprophagous generalist and is among the first and most abundant species at mammal carrion in forests in Peninsular Malaysia [19]. Blowflies also potentially concentrate faecal DNA while feeding coprophagously [20,13].

One key factor affecting successful detection of ingested DNA is the digestion efficiency of the hematophagous, coprophagous or saprophagous feeder [13]. Knowledge of the persistence period of amplifiable mammal DNA in blowfly guts is essential for designing standardized trapping methods (e.g. [21]) for large-scale mammal monitoring via this approach. Typically, for ecological studies, blowflies are captured at baited traps, and may remain alive in the trap for hours [22] or days [23] before collection by the researcher. Forensic studies have determined the post-feeding detection period of human mtDNA in the guts of blowfly maggots (*Calliphora vicina*) to be between 24 h and 48 h [24]. However, the post-feeding persistence period of mammal DNA in the guts of adult blowflies still lacks any reliable data.

**Fig 1:**
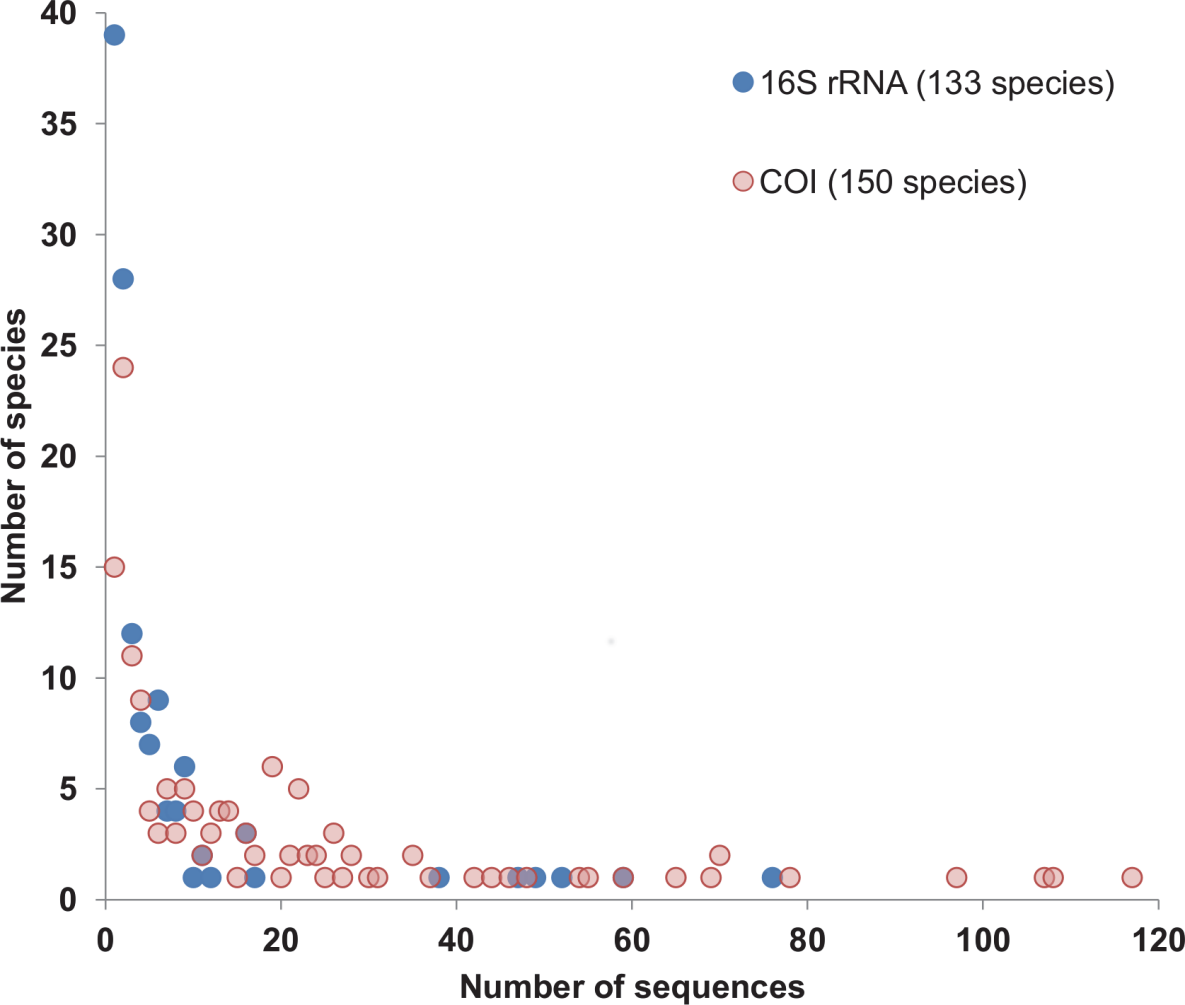
Number of *16S rRNA* and *COI* sequences publicly available on GenBank for the 246 mammal species (excluding *Homo sapiens*) found in Peninsular Malaysia.

A second key factor for the success of invertebrate-derived DNA approaches is selection of the target DNA region. The region must be easy to PCR amplify from taxonomically unknown samples, must be variable among taxa to permit species identification, and reference sequences of known species must exist in order to match the amplified fragment. Previous studies have used *16S rRNA* and *12S rRNA* for detection of mammal mtDNA from blowflies [14], *16S rRNA* from leeches [15], *cytochrome b* from mosquitoes and sandflies [25,26] and *cytochrome c oxidase I* (*COI*) from ticks and tsetse flies [17,27]. A fragment of *COI* is a preferred target for a number of reasons. Variation in *COI* has been used successfully to discriminate and identify mammals in Southeast Asia [28]. There are more *COI* sequences than *16S rRNA* sequences on GenBank for mammal species (after excluding *Homo sapiens* sequences) (Fig 1) and this includes BARCODE standard records [29]. Therefore we suggest the chance of accurately assigning an unknown mammal to a species is higher for a fragment of *COI* than for other gene regions. Broad “universal” primers have been designed for amplification of mammal *COI* barcodes [30] but a short target is required for ingested DNA, which is likely to be partially degraded and difficult to amplify [13]. Considering that field-application will likely involve pooling a large number of blowflies for cost-effective high-throughput sequencing [31], a 100–300 bp fragment is preferred [32]. A “universal DNA mini-barcode for biodiversity analysis” has been published previously [33] but the primers have variable success amplifying mammal species (77% as reported by Meusnier *et al*. [33]; 0% as determined *in silico* by Ficetola *et al*. [34]; 80% as reported by Arif *et al*. [35]) and also will amplify blowfly DNA, which is likely to be present at higher concentration.

As a step towards development of blowfly-derived DNA as an effective method for mammal monitoring in a biodiversity hotspot, Peninsular Malaysia, our objectives were (i) to determine the persistence period of amplifiable mammal mtDNA in blowfly guts through a laboratory feeding experiment, and (ii) to design and test primers that can selectively amplify mammal DNA mini-barcodes in the presence of high concentrations of blowfly DNA.

## Materials and Methods

### Ethics statement

Our protocol for minimally-invasive collection of mammal DNA samples (hair, wing punches) has been approved by the University of Malaya Institutional Animal Care and Use Committee (UMIACUC) (Ref. ISB/02/1212013/JJW (R)) and the Department of Wildlife and National Parks, Peninsular Malaysia (Ref. JPHL&TN(IP): 80-4/2 Jld16(24)). No specific permits were required, however, approval was obtained from the land owners/managers for animal (mammal and blowfly) sampling and no protected species were sampled.

### Persistence of mammal mtDNA in blowfly guts

To obtain a sample of blowflies of known age, physiological state, and feeding history a laboratory culture of *Chrysomya megacephala* was established. A rotting fish carcass was obtained from a local supermarket and placed outside the Museum of Zoology, University of Malaya, Kuala Lumpur, to encourage egg deposition by wild blowflies. The fish was then moved into a 39 x 25 x 33 cm sealed plastic container and the hatching larvae provided with more fish until pupation. Once all pupae had emerged *C*. *megacephala* adults were selected out (based on the morphological characters of the species) and sorted into three containers of approximately 100 blowflies each.

The adult blowflies were then starved for 24 h to allow digestion of any food taken and to adjust to similar hunger levels. After the starvation period pieces of market-supplied beef liver (*Bos taurus*) were placed into the containers for 4 h (06:00–10:00). We observed that all blowflies fed almost immediately upon provision of food. After the beef liver was removed sugar water was provided as the only food source. At 8, 16, 24, 48, 72, 96, 120, 144 h following removal of the beef liver, 9 individual, adult blowflies were selected arbitrarily (3 from each container) at each interval and were frozen at -20°C. The blowfly’s guts were then dissected out with sterile implements for DNA extraction using NucleoSpin Tissue kit (Macherey-Nagel, Germany), following the manufacturer’s instructions. To provide positive and negative controls respectively, DNA was also extracted directly from the market-supplied beef liver and wild-caught blowfly legs (*Chrysomya megacephala*).

PCR was performed using beef-specific primers targeting a 75 bp region of the *Bos taurus cytochrome b* mtDNA (BSP F: 5’-CCCGATTCTTCGCTTTCCAT-3’and BSP R: 5’-CTACGTCTGAGGAAATTCCTGTTG-3’) [36]. FastMix Frenche Hot Start PCR pre-mix (Intron Biotechnology, Korea) was used for all PCR reactions, adding 1 μL of each primer and 1 μL of DNA extract. The thermal cycling conditions were 94°C for 3 min, followed by 30 cycles of 94°C for 30 s, 54°C for 30 s, 72°C for 1 min and a final extension at 72°C for 5 min. PCR products were visualised on a 1.5% agarose gel stained with 1 x GelRed (Biotium, USA). We also created beef/blowfly DNA mixtures from the control DNA extracts, and performed PCR to determine relative concentrations of beef DNA based on visual comparison of the electrophoresis images.

### Design and testing of mammal primers

We assembled a test dataset of 41 DNA extracts from 41 mammal species (16% of the mammal species found in Peninsular Malaysia) collected during our previous field sampling in Peninsular Malaysia [37–39] and from collection at Ulu Gombak Forest Reserve Selangor, Gerik Perak, and nearby the Museum of Zoology, University of Malaya, Kuala Lumpur (see S1 Dataset). Based on our exploration of PCR amplification of *COI* with this dataset and other mammal samples, we have found that Uni-Mini-bar F [33] and RonM [30] have good success as a forward primers, but LepF1 [40], HCO2198 [41], and VF1d [30] have lower success. We have found that Uni-Mini-bar R [33] has low success as a reverse primer, but VR1d [30] has good success. Given the high success of Uni-Mini-bar F and RonM, and the 210 bp distance between these primers, we proceeded to design a reverse primer targeting the RonM binding site.

**Fig 2:**
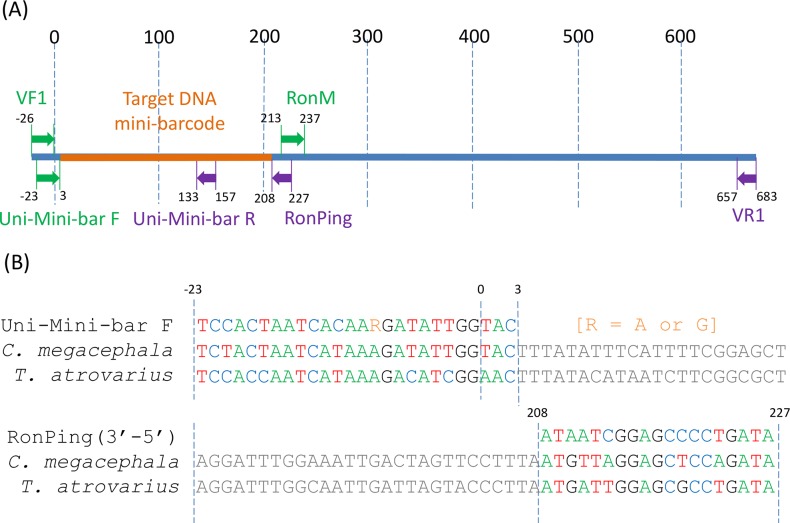
The binding sites of the primers were in relation to a *Chrysomya megacephala* (blowfly) and *Thomomys atrovarius* (Smooth-toothed pocket gopher) sequence. A) Relative positions of mammal primers on the *COI* barcode region. B) The binding sites of the primers Uni-Mini-bar F and RonPing (reverse complement).

We aligned mammal *COI* sequences retrieved from GenBank and used the program Primer3 [42] to select a 19 bp region slightly upstream of RonM, which enabled design of a reverse primer with appropriate physical and structural properties. When used in combination with Uni-Mini-bar F, the new primer RonPing (5'-tatcaggggctccgattat-3') should amplify a 205 bp fragment at the 5’ end of the *COI* barcode region (Fig 2).

The success of Uni-Mini-bar F/ RonPing and Uni-Mini-bar F/ Uni-Mini-bar R across mammal species was then systematically compared using our 41 species dataset. PCR was performed using FastMix Frenche Hot Start PCR pre-mix (Intron Biotechnology, Korea) and COI Fast thermocycling program [43] for all reactions, with slight modification to the DNA volume (0.5–2 μL) depending on DNA extraction method.

Next we tested the ability of the Uni-Mini-bar F/ RonPing combination to amplify low concentrations of mammal DNA in the presence of high concentrations of blowfly DNA. We first confirmed (through testing on multiple DNA extractions with various thermocycling conditions) that the primer combination does not amplify *Chrysomya megacephala COI*. We then mixed DNA extracts from ten mammal species with DNA extracts from blowfly legs in a ratio of 1 part mammal (~0.43 ng) to 16 parts blowfly (~7.0 ng). PCR was performed for the mixed DNA samples as described above. Additionally, we used DNA extracts from the 48 h post-feeding samples from the feeding experiment above as templates for PCR with Uni-Mini-bar F/ RonPing.

A further test involved the detection of mammal DNA from wild-caught blowflies. Four baited traps (modified from [13]) were set at Rimba Ilmu, University of Malaya (S1 Fig). Rimba Ilmu is an 80 ha botanical garden and a habitat for small mammals such as bats, squirrels, tree shrews and rats. The traps were emptied every 24 h and blowflies were frozen at -20°C. The guts of 30 blowflies were then dissected out with sterile implements for DNA extraction and PCR with Uni-Mini-F/ RonPing as above. PCR products were sequenced in both directions by a local company (MYTACG-Kuala Lumpur, Malaysia). DNA sequences are available on BOLD in the dataset DS-RONPING (http://www.boldsystems.org/index.php/Public_SearchTerms?query=DS-RONPING).

To evaluate the success of the 205 bp DNA mini-barcode amplified by Uni-Mini-bar F/ RonPing for species assignment, *COI* sequences from mammal species found in Malaysia (based on [44]) were mined from GenBank. We retrieved *COI* sequences >600 bp and the sequences were trimmed to the 205 bp DNA mini-barcode target amplified by Uni-Mini-bar F/ RonPing. A neighbor-joining tree based on number of differences was produced from the aligned sequences using MEGA 6.0 [45].

## Results

### Persistence of mammal mtDNA in blowfly guts

Blowfly (*Chrysomya megacephala*) guts sampled at 24 h post-feeding, or earlier, all contained amplifiable beef mtDNA (S2 Fig). At 48 h post-feeding 78% of blowfly guts had amplifiable beef mtDNA, but this dropped sharply to 44% at 72 h and 22% at 96 h. At these later times, when amplification was successful, the bands were fainter indicating lower concentrations of DNA. There was no successful amplification from guts sampled at 120 h post-feeding and later.

### Design and testing of mammal-specific primers

Our newly designed RonPing primer had a low number of mismatches (less than six) with most of the mammal *COI* sequences retrieved from GenBank (S3 Fig). RonPing had five mismatches with *Chrysomya megacephala* (Fig 2) and did not amplify this species. *Canis lupus* (Order: Carnivora) also had five mismatches located in approximately the same positions, with one near to 3’end of primer, but *Canis lupus COI* was amplified with RonPing. Other mammal species showing 3–4 mismatches were also amplified and sequenced successfully.

**Fig 3:**
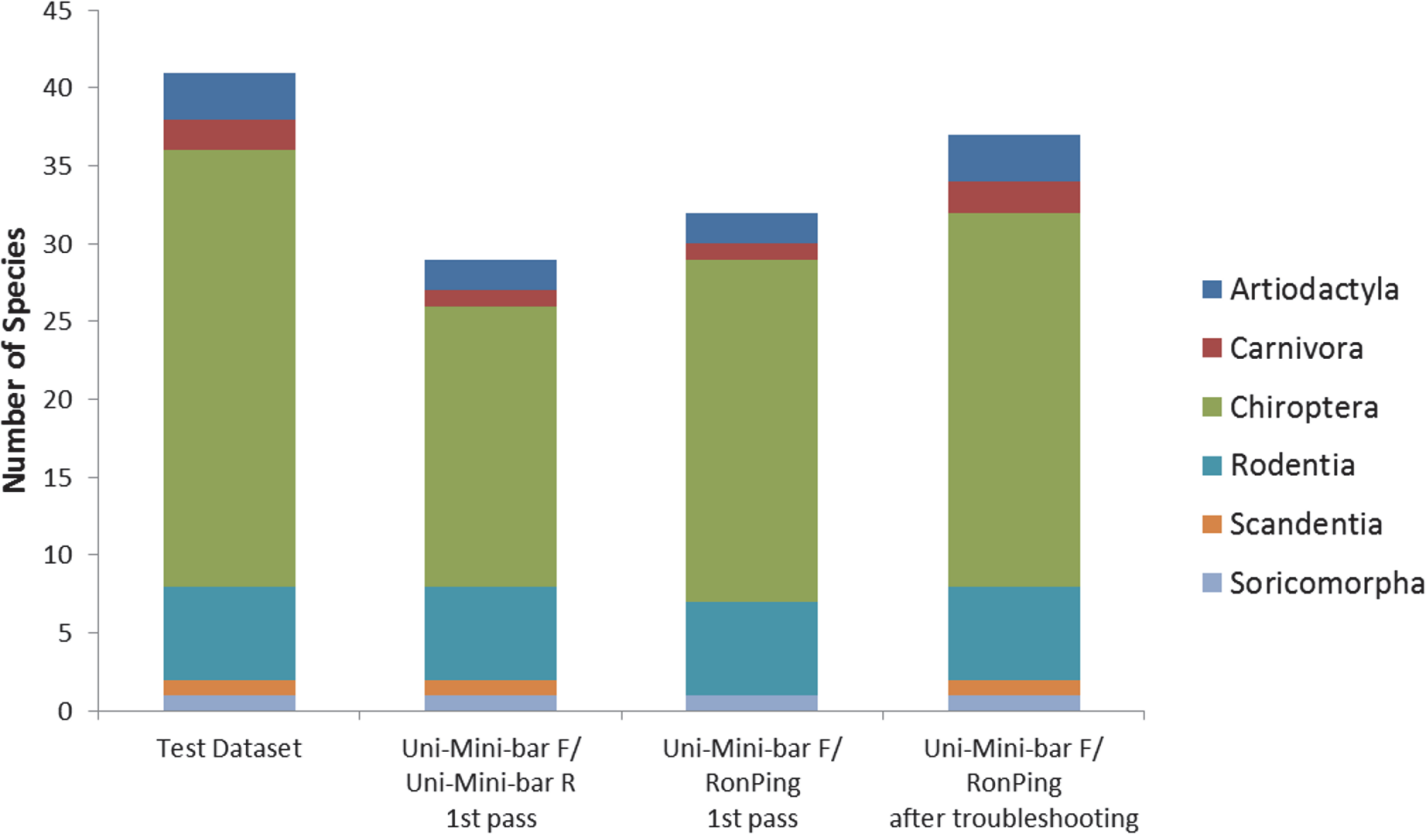
Comparison of amplification success using primer pairs: Uni-Mini-bar F/ RonPing and Uni-Mini-bar F/ Uni-Mini-bar R for Artiodactyla (n = 3), Carnivora (n = 2), Chiroptera (n = 28), Soricomorpha (n = 1), Rodentia (n = 5) and Scandentia (n = 1).

The success of primer pairs, Uni-Mini-bar F/ Uni-Mini-bar R [33] and Uni-Mini-bar F/ RonPing in amplifying *COI* from 41 mammal species extracts were 71% and 78% respectively (Fig 3). A further round of PCR using Uni-Mini-bar F/ RonPing yielded a higher proportion of species amplified (89%). Mammal sequences amplified from ten mammal/*C*. *megacephala* DNA mixtures using Uni-Mini-bar F/ RonPing had high quality peaks and were clear of contamination (S4 Fig). The Uni-Mini-bar F/ RonPing combination showed successful amplification from 27% of wild-caught *C*. *megacephala* with sequenced amplicons showing close matches (<95%) to *Rhinolophus* sp., *Bos taurus* and *Gallus gallus*.

Examining the target 205 bp region among mammals from Peninsular Malaysia mined from GenBank (113 species), all species, except for seven, possessed a unique *COI* sequence or unique sets of *COI* sequences for the 205 bp DNA mini-barcode (S5 Fig). The region was also able to separate 26 “dark” bat taxa recognized in previous DNA barcoding studies (e.g. *Balionycteris maculate*, *Hipposideros armiger* and *Myotis muricola*) [28].

## Discussion

Calvignac-Spencer and colleagues [14] demonstrated the potential of sequencing invertebrate-derived DNA for mammal monitoring and suggested the advantage of blowflies over other invertebrates [13]. However, certain questions remained regarding the field-application in large-scale mammal monitoring. Blowflies have been trapped in a variety of ways depending on the purpose of sampling (e.g. for veterinary purposes [23, 46]). Calvignac-Spencer and colleagues [14] sampled flies opportunistically immediately upon arrival at bait. A probable field-application scenario for large-scale mammal monitoring would see a large number of baited traps, where the trapped blowfly is unable to make contact with the bait and is kept alive until collection (e.g. [47]). The traps would be set for a number of hours before the researcher is able to return to empty them. In field experiments *Chrysomya megacephala* usually arrived to fresh carrion within 24 h of exposure [19] meaning blowflies could potentially be in a trap for a number of hours digesting any mammal DNA already present in their guts. Our results indicate amplifiable mammal mtDNA persists in the guts of adult *C*. *megacephala* for 24 h—96 h post-feeding (89% at 48 h) which is consistent with that determined for blowfly maggots [24] and other dipterans (mosquitoes, tsetse flies) [48]. In contrast, mammal DNA can persist in ticks and leeches for at least several months [15, 48]. The longer persistence period may seem like an advantage to using these taxa but means it is more difficult to determine when the detected mammal species was present at the sampling site. Based on our findings we suggest that blowflies will need to be retrieved from traps and processed at least every 24 h to maximize the chance of amplifying mammal DNA from their previous meal. The interval between a blowfly feeding and looking for its next meal is unknown, but would also be a factor affecting the successful detection of ingested DNA. The high success in detecting mammal DNA from wild-caught blowflies (27% of blowflies contained detectable vertebrate DNA, with multiple species detected in some individuals) trapped over a period of 24 hours indicates the potential of a standardized trapping protocol with retrieval of blowflies every 24 h for effective mammal monitoring in the field.

Here, we provide the first experimental indication that successful detection of mammal DNA from blowflies is due to mammal DNA in their guts as opposed to mammal DNA being carried on their exoskeleton as a result of landing on mammal tissues or faeces. This was not addressed directly in previous studies [14]. The gradual decline in amplifiable mammal DNA could indicate a lack of severe enzymatic breakdown of ingested DNA in the fore-gut. For blowflies primary digestion is achieved by secretion of salivary enzymes onto food before it is ingested orally [24, 49]. The persistence period of amplifiable DNA may be different depending on the length of the target fragment chosen for amplification. It is worth to note that our experiment to assess mammal DNA persistence is likely to provide an upper estimate as the substrate the blowflies were fed with was of high quality (beef liver) and energy expenditure was limited (flies were kept in boxes). We found no difference between amplification success using the beef-specific primers targeting 75 bp and amplification success using Uni-Mini-bar F/ RonPing targeting 205 bp.

The use of *16S rRNA* by Calvignac-Spencer and colleagues [14] was problematic. While the *16S rRNA* fragment is easy to amplify using primers with broad taxonomic coverage across Mammalia, the short “barcode” produced has low species resolution i.e. multiple closely related species can share the same haplotype [34]. Our results suggest targeting *COI*, the animal DNA barcode [50], is a very practical option, providing the chance to exploit the identification capacity of BOLD [51] and the well-characterised patterns of species level divergence at this region (e.g. [28]). The new primer, RonPing, when used in combination with Uni-Mini-bar F amplifies a 205 bp fragment of *COI*. This appears to be an optimal length for a DNA mini-barcode allowing amplification from degraded samples, such as invertebrate-derived DNA, while not suffering a reduction in the ability to distinguish species [33]. This target also falls within the maximum read length for high-throughput sequencing (e.g. 300 bp for the Illumina MiSeq [32]), including spare length for MID tags to separate multiple samples [52].

The new primer combination was able to amplify a higher proportion of mammal species than previously proposed combinations [33]. Another significant advantage of the Uni-Mini-bar F/ RonPing combination is the fact that it does not amplify *Chrysomya megacephala COI*, even when the ratio of blowfly DNA to mammal DNA is high. This is likely due to RonPing having a double mismatch with *C*. *megacephala* within 5 bp of the 3’ end including a purine-purine mismatch (A-G) [53]. Sixteen mammal species also had an A-G mismatch in the same position, but they only had one mismatch within 5 bp from 3’end of primer not the *C*. *megacephala* double, which may be the main reason for successful amplification from these DNA templates. Lack of *C*. *megacephala* amplification negates the need for blocking primers [54] or, if no blocking probes are used in high-throughput sequencing, prevents significant wastage of sequencing effort due to amplification and sequencing of blowfly DNA [55]. The low number and even distribution of mainly pyrimidine-pyrimidine and purine-pyrimidine mismatches between the RonPing primer and GenBank mammal sequences suggest primer bias might not be too severe and consequently the primer may produce a less biased estimate of DNA templates present [53]. Although the ecoPCR program [34] predicted that the Uni-Mini-bar F/ RonPing combination could only amplify 19% of mammal species by allowing three mismatches to the whole mitochondrial genomes on GenBank (results not shown), results coherent with *in vitro* PCR can be obtained by allowing a higher number of mismatches.

The 205 bp *COI* fragment was successful in distinguishing nearly all examined mammal species from Peninsular Malaysia and separating “dark” bat taxa, previously recognised species that lack formal taxonomic status [39]. This suggested the potential of detecting cryptic taxa overlooked by traditional methods [28]. The species which shared haplotypes at the 205 bp region such as *Rattus tiomanicus* and *R*. *rattus* included sequences which have previously been identified as problematic and most likely represent misidentifications or contamination rather than shared haplotypes [56].

## Conclusion

Our findings suggest our new DNA mini-barcode target and a standardized trapping protocol with retrieval of blowflies every 24 h could point the way forward in the development of blowfly-derived DNA as an effective method for mammal monitoring in Peninsular Malaysia. The next step would be a comprehensive comparison of diversity measures for mammals produced by the blowfly-derived DNA approach and by traditional monitoring approaches such as cage traps, mist nets, hair traps, camera traps, or scat samples.

## Supporting Information

S1 Dataset List of 41 mammal specimens from Malaysia used for comparison of amplification success between primer pairs, Uni-Mini-bar F/ RonPing and Uni-Mini-bar F/ Uni-Mini-bar R. (PDF)

S1 Fig Baited blowfly trap for mammal monitoring. (PDF)

S2 Fig PCR amplification of beef mtDNA from blowfly guts post feeding (8 h—120 h). (PDF)

S3 Fig Number of mismatches between the RonPing primer sequence (19 bp) and 1472 mammal *COI* sequences from GenBank. (PDF)

S4 Fig Examples of fragments of mammal sequences amplified using Uni-Mini-bar F/ RonPing primers from mammal/*C*. *megacephala* DNA mixtures. (PDF)

S5 Fig Neighbor-joining tree of 205 bp *COI* sequences for mammal species from Peninsular Malaysia from GenBank. Solid triangles represent clusters of multiple conspecifics.
(PDF)
