# Supplementary material for: Reading Mammal Diversity from Flies: The Persistence Period of Amplifiable Mammal mtDNA in Blowfly Guts (Chrysomya megacephala) and a New DNA Mini-Barcode Target
Source: PLoS One. 2015 Apr 21;10(4):e0123871. doi: 10.1371/journal.pone.0123871 (PMC4405593; doi:10.1371/journal.pone.0123871)
Supplement: S1 Dataset — (PDF) [file pone.0123871.s001.pdf]

Dataset S1. List of 41 mammal species from Malaysia for comparison of amplification success between primer pairs, Uni-Mini-bar F/RonPing and Uni-Mini-bar F/Uni-Mini-bar R and BOLD Process ID numbers for those sequenced after troubleshooting.

| Specimen Number | Species | Uni-Mini-bar F/ Uni-Mini-bar R 1st Pass | Uni-Mini-bar F/ RonPing 1st Pass | Uni-Mini-bar F/ RonPing Troubleshooting |
|---|---|---|---|---|
| BT31B | *Balionycteris maculata* | / | / | RONP010-14 |
| P16B | *Bandicota indica* | / | / | RONP029-14 |
| BF5A | *Bos taurus* | × | × | RONP001-14 |
| DOGB | *Canis lupus familiaris* | × | × | RONP031-14 |
| GOATB | *Capra hircus* | / | / | RONP050-14 |
| BGM19A | *Chironax melanocephalus* | × | / | RONP002-14 |
| BK28B | *Cynopterus brachyotis* | / | / | RONP049-14 |
| BT4B | *Cynopterus horsefieldii* | × | / | RONP040-14 |
| BK4B | *Cynopterus* JLE sp.A | × | / | RONP012-14 |
| BT73B | *Eonycteris spelaea* | / | / | RONP025-14 |
| CATB | *Felis catus* | / | / | RONP027-14 |
| BT64A | *Glischropus tylopus* | / | / | RONP009-14 |
| TI13B | *Hipposideros bicolor131* | / | × | RONP033-14 |
| T25B | *Hipposideros cervinus* | × | / | RONP026-14 |
| T27B | *Hipposideros* cf. *larvatus* | / | / | RONP015-14 |
| BK1B | *Hipposideros diadema* | × | × | RONP034-14 |
| BT30B | *Hipposideros doriae* | / | / | RONP042-14 |
| BK13B | *Kerivoula* cf. *hardwickii* | / | / | RONP019-14 |
| BT25B | *Kerivoula minuta* | / | / | RONP039-14 |
| BT44B | *Kerivoula papillosa* | / | / | RONP014-14 |
| BT17A | *Kerivoula pellucida* | / | / | RONP004-14 |
| PING14B | *Leopoldamys sabanus* | / | / | RONP035-14 |
| BK12B | *Macroglossus sobrinus* | / | / | RONP018-14 |
| PING15B | *Maxomys surifer* | / | / | RONP036-14 |
| BK22A | *Megaderma lyra* | / | / | RONP005-14 |
| BT59B | *Megaderma spasma* | × | × | × |
| BT50B | *Megaerops ecaudatus* | / | / | RONP041-14 |
| T74B | *Murina aenea* | / | / | RONP016-14 |
| BT10B | *Murina cyclotis* | × | / | RONP013-14 |
| BK32B | *Murina suilla* | × | × | × |
| AF1B | *Mus musculus* | / | / | RONP051-14 |
| BT2B | *Myotis muricola* | / | / | RONP037-14 |
| PING18A | *Rattus andamanensis* | / | / | RONP006-14 |
| PING3B | *Rattus tiomanicus* | / | / | RONP038-14 |
| BT1B | *Rhinolophus acuminatus* | × | × | × |
| BK49B | *Rhinolophus affinis* | / | / | RONP021-14 |
| BT34B | *Rhinolophus trifoliatus* | × | × | × |
| BK67B | *Rhinolophus yunanensis* | / | / | RONP011-14 |
| Z2B | *Suncus murinus* | / | / | RONP032-14 |
| WB8B | *Sus scrofa* | / | / | RONP008-14 |
| PING11B | *Tupaia glis* | / | × | × |
| | Total | 29/41 (71%) | 32/41 (78%) | 36/41 (89%) |
