# Supplementary figures and images for: Reading Mammal Diversity from Flies: The Persistence Period of Amplifiable Mammal mtDNA in Blowfly Guts (Chrysomya megacephala) and a New DNA Mini-Barcode Target
Source: PLoS One. 2015 Apr 21;10(4):e0123871. doi: 10.1371/journal.pone.0123871 (PMC4405593; doi:10.1371/journal.pone.0123871)

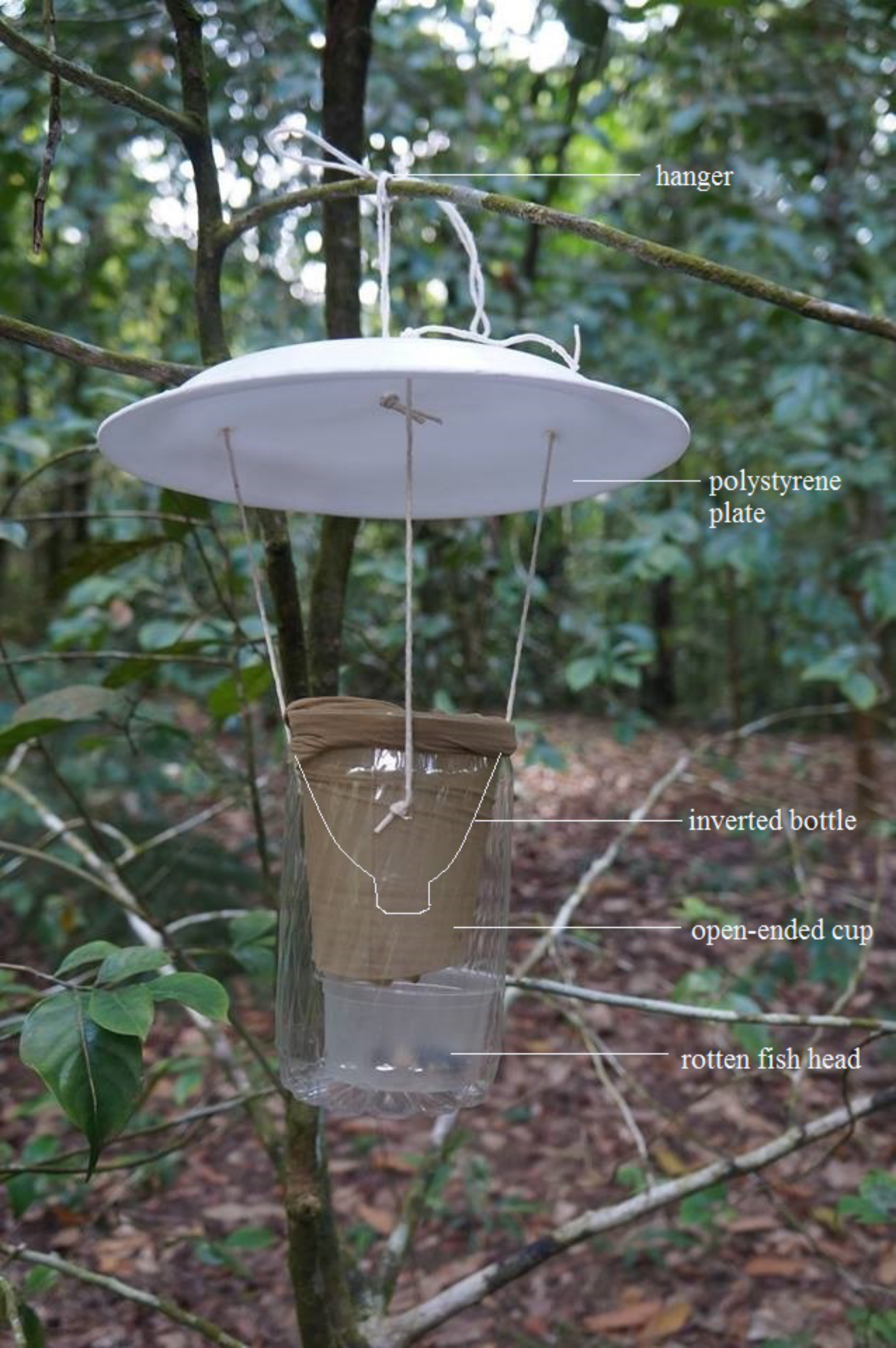

hanger

polystyrene  
plate

inverted bottle

open-ended cup

rotten fish head

Supplement: S1 Fig — (PDF) [file pone.0123871.s002.pdf]

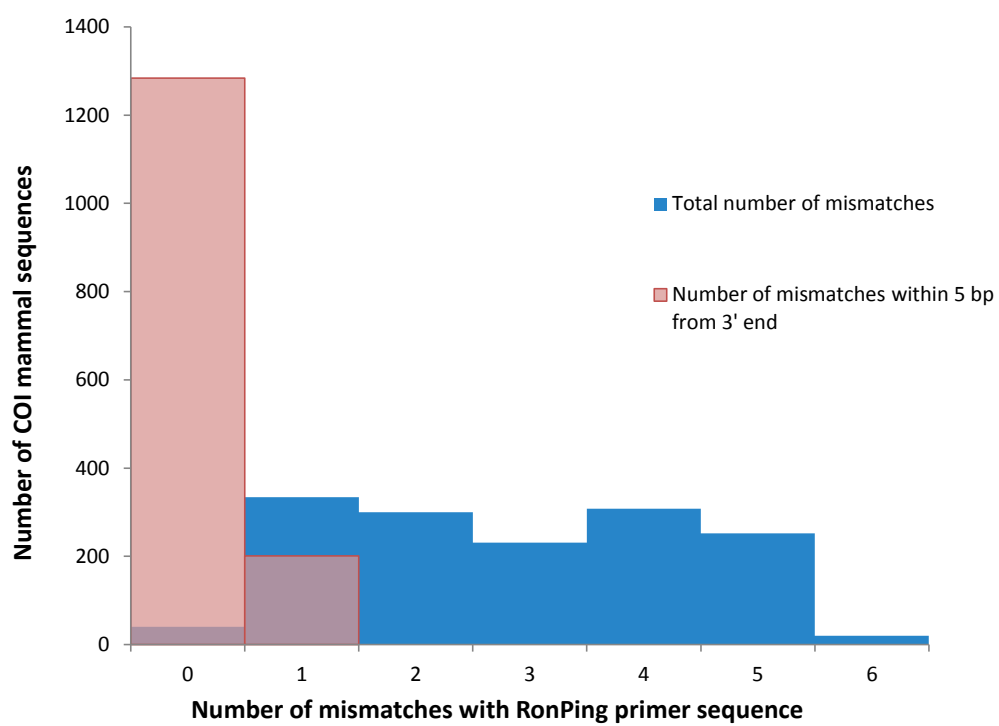

Supplement: S3 Fig — (PDF) [file pone.0123871.s004.pdf]

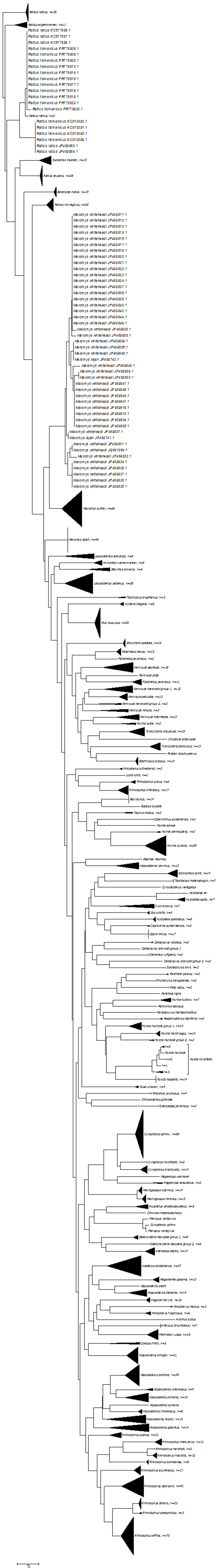

Supplement: S5 Fig — Solid triangles represent clusters of multiple conspecifics. (PDF) [file pone.0123871.s006.pdf]
