# Supplementary material for: Reading Mammal Diversity from Flies: The Persistence Period of Amplifiable Mammal mtDNA in Blowfly Guts (Chrysomya megacephala) and a New DNA Mini-Barcode Target
Source: PLoS One. 2015 Apr 21;10(4):e0123871. doi: 10.1371/journal.pone.0123871 (PMC4405593; doi:10.1371/journal.pone.0123871)
Supplement: S2 Fig — (PDF) [file pone.0123871.s003.pdf]

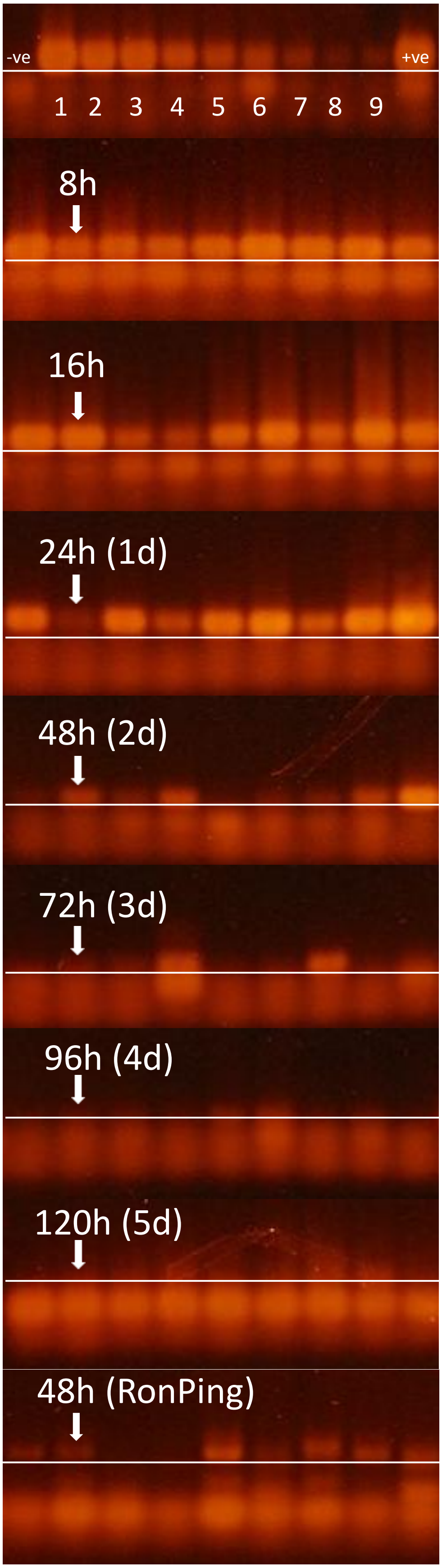

The top row 1-9 shows amplification of beef DNA from mixed blowfly/beef DNA extracts at different concentrations. The amount of beef DNA in the PCR was: 1.25 ng, 0.83 ng, 0.50 ng, 0.28 ng, 0.15 ng, 0.08 ng, 0.04 ng, 0.02 ng and 0.01 ng for 1-9 respectively. The -ve control is DNA extracted from a wild-caught blowfly leg and the +ve control is DNA extracted from beef liver directly. The last row shows amplification using Uni-Mini-bar F/ RonPing primers at 48 h post feeding.
