# Supplementary material for: Reading Mammal Diversity from Flies: The Persistence Period of Amplifiable Mammal mtDNA in Blowfly Guts (Chrysomya megacephala) and a New DNA Mini-Barcode Target
Source: PLoS One. 2015 Apr 21;10(4):e0123871. doi: 10.1371/journal.pone.0123871 (PMC4405593; doi:10.1371/journal.pone.0123871)
Supplement: S4 Fig — (PDF) [file pone.0123871.s005.pdf]

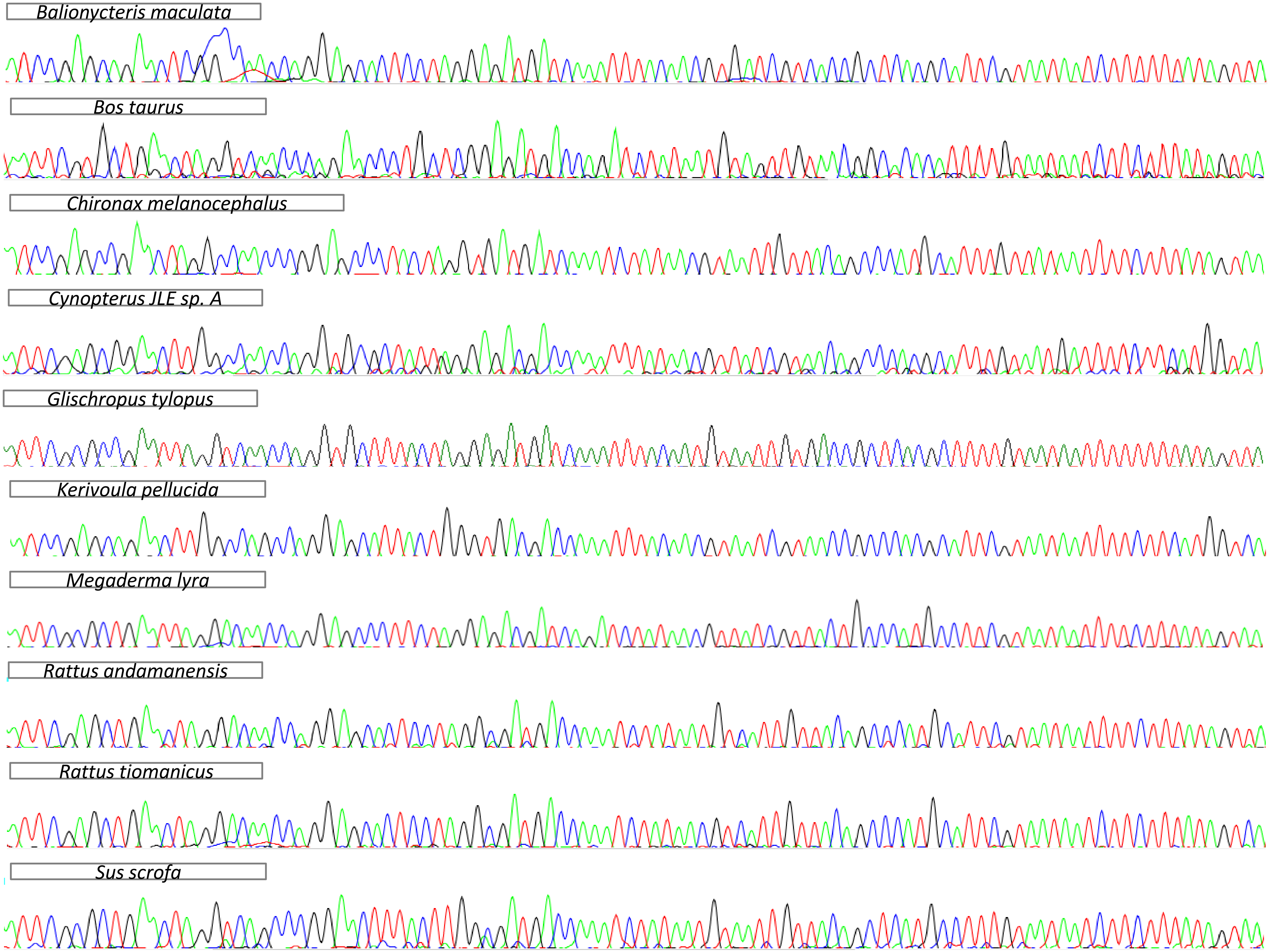

Balionycteris maculata
Bos taurus
Chironax melanocephalus
Cynopterus JLE sp. A
Glischropus tylopus
Kerivoula pellucida
Megaderma lyra
Rattus andamanensis
Rattus tiomanicus
Sus scrofa
